# Prevalence of Human Papillomavirus in 5,072 Consecutive Cervical SurePath Samples Evaluated with the Roche Cobas HPV Real-Time PCR Assay

**DOI:** 10.1371/journal.pone.0059765

**Published:** 2013-03-22

**Authors:** Sarah Preisler, Matejka Rebolj, Anette Untermann, Ditte Møller Ejegod, Elsebeth Lynge, Carsten Rygaard, Jesper Bonde

**Affiliations:** 1 Department of Pathology, Copenhagen University Hospital, Hvidovre, Denmark; 2 Clinical Research Centre, Copenhagen University Hospital, Hvidovre, Denmark; 3 Department of Public Health, University of Copenhagen, Copenhagen, Denmark; Centers for Disease Control and Prevention, United States of America

## Abstract

New commercially available Human Papillomavirus (HPV) assays need to be evaluated in a variety of cervical screening settings. Cobas HPV Test (cobas) is a real-time PCR-based assay allowing for separate detection of HPV genotypes 16 and 18 and a bulk of 12 other high-risk genotypes. The aim of the present study, Horizon, was to assess the prevalence of high-risk HPV infections in an area with a high background risk of cervical cancer, where women aged 23–65 years are targeted for cervical screening. We collected 6,258 consecutive cervical samples from the largest cervical screening laboratory in Denmark serving the whole of Copenhagen. All samples were stored in SurePath media. In total, 5,072 samples were tested with cobas, Hybrid Capture 2 High Risk HPV DNA test (HC2) and liquid-based cytology. Of these, 27% tested positive on cobas. This proportion decreased by age, being 43% in women aged 23–29 years and 10% in women aged 60–65 years. HC2 assay was positive in 20% of samples, and cytology was abnormal (≥ atypical squamous cells of undetermined significance) for 7% samples. When only samples without recent abnormalities were taken into account, 24% tested positive on cobas, 19% on HC2, and 5% had abnormal cytology. The proportion of positive cobas samples was higher than in the ATHENA trial. The age-standardized cobas positivity vs. cytology abnormality was 3.9 in our study and 1.7 in ATHENA. If in Copenhagen the presently used cytology would be replaced by cobas in women above age 30 years, an extra 11% of women would based on historical data be expected to have a positive cobas test without an underlying cervical intraepithelial lesion grade 3 or worse. Countries with a high prevalence of HPV infections should therefore proceed to primary HPV-based cervical screening with caution.

## Introduction

The higher sensitivity for high-grade cervical intraepithelial neoplasia (CIN) of Human Papillomavirus (HPV) testing compared with cytology [1] could protect more women from developing cervical cancer [2], [3]. Because of this, it is expected that HPV testing will slowly replace cytology in primary cervical screening. So far, HPV DNA testing has been implemented into primary cervical screening e.g. in the USA, Mexico, and the Spanish region of Castile and Leon, whereas the Netherlands made a recommendation for primary screening in 2011. In other countries, HPV testing has been used in triage of women aged ≥30 years with atypical squamous cells of undetermined significance (ASCUS) for colposcopy, and in surveillance after CIN treatment.

For several years, HPV DNA testing has been synonymous with Qiagen’s *digene* Hybrid Capture 2® High Risk HPV DNA test (HC2; Qiagen, Gaithersburg, MA, USA). The HC2 assay has been extensively studied all over the world in randomized controlled trials and numerous split-sample studies [1], [4], [5]. Because of this, it is widely considered a standard HPV DNA assay [6]. Recently, more HPV assays have become commercially available. The designs of these assays differ from that of HC2 in terms of the targeted viral genes and of the testing methods. Accordingly, their clinical characteristics may differ from those of HC2 and need to be evaluated in a variety of settings [7].

The cobas® HPV Test (cobas; Roche Diagnostics, Pleasanton, CA, USA), approved by the USA Food and Drug Administration in 2011, is a fully automated real-time polymerase chain reaction (PCR) assay. The assay batches up to 94 samples together with positive and negative controls. It allows for a differentiated positive result distinguishing HPV genotypes 16 and 18 separately next to the bulk of 11 other high-risk and one possibly high-risk HPV genotypes (31, 33, 35, 39, 45, 51, 52, 56, 58, 59, 66 and 68) [8]. Because genotypes 16 and 18 alone cause about 70% of all cervical cancers [9], use of HPV16/18 genotyping has been proposed as a triage procedure for referral of HPV-positive, cytology-normal women for colposcopy [10]. The largest study to date evaluating the cobas test has been ATHENA, a split-sample study undertaken in 46,887 women aged ≥21 years presenting for routine cervical screening at 61 clinical sites in the USA [11]. In ATHENA, cobas testing in women aged ≥25 years was almost 40% more sensitive than cytology for detection of ≥CIN3 (crude sensitivities 92% vs. 53%) [12]. Moreover, referral for colposcopy of cytology-normal/cobas-positive women with HPV16 or HPV18 had a good positive predictive value for detecting ≥CIN3 [13]. Several smaller studies evaluated the use of cobas HPV testing in women recommended for further follow-up owing to abnormal screening tests [14]–[18]. All of these studies were undertaken using samples stored in PreservCyt, universal collection medium, or specimen transport medium.

In the present population-based split-sample study, we compared cobas testing with liquid-based cytology (LBC) and HC2 on consecutive samples from a large cervical cytology laboratory covering all of Copenhagen, Denmark. The population from this area is well-screened but has a high background risk of cervical cancer. In 1958–62, prior to the start of screening, the age-standardized incidence of cervical cancer in Copenhagen was 35 per 100,000 (world standard population) [19]. The Copenhagen samples in the present study were stored in SurePath® media (BD Diagnostics–TriPath, Burlington, NC, USA).

## Materials and Methods

### Study Population

In Denmark, women aged between 23 and 49 years without a smear or a biopsy within the last three years are personally invited for cytology-based cervical screening, as are women aged 50–65 years if they had no screening in the last five years. In 2010, about 76% of women were screened within the recommended interval [20]. The Department of Pathology, Hvidovre University Hospital, is the largest cervical cytology laboratory in Denmark receiving 66,000 LBC samples a year (2011). The laboratory handles all primary and follow-up cervical samples from women living in Copenhagen, which includes the Copenhagen and Frederiksberg municipalities.

During the period from 10 June 2011 to 25 August 2011, a total of 12,138 routine cervical samples were received at the laboratory. On date of arrival, the samples were registered in the national Pathology Data Bank using the Danish personal identification number (CPR number) which includes the date of birth. Samples were placed in racks of 48, and labeled with the woman’s CPR number, a laboratory specimen identifier, and a barcode.

The present study, Horizon, was nested into the routine laboratory practice. It utilized the residual material left in the vial after SurePath-based LBC had been completed, and after any postquot HC2 triage testing of samples with ASCUS diagnoses in women aged ≥30 years. A total of 6,258 samples (52%) were selected for the present study, taking the lowest rack numbers of samples on testing days equally from Monday to Friday. Based on capacity and processing considerations at the molecular biology laboratory, the target number of samples was set to 5,000. A maximum of 192 samples (four racks) were processed per day. Samples with insufficient residual volume for further HPV testing (n = 1,165) were excluded from the study. An additional 21 samples were excluded for technical reasons due to human error. The final analysis of cobas testing could therefore be undertaken on 5,072 samples (81% of those selected for the study). In concordance with the protocol reviewed by the manufacturers prior to the study, 2 ml of SurePath media was added to the available residual material of approximately 2 ml (dilution factor roughly 1∶1), in order to obtain enough volume for additional testing which will be reported separately. All testing was done in the same laboratory.

In total, 5,013 samples (98.8%) were from unique women, whereas the remaining 59 (1.2%) were collected from 29 women. Women’s screening history from 1 January 2000 onwards was ascertained from the nation-wide Danish Pathology Data Bank [21]. Study samples with an earlier diagnosis of cervical cancer, or a CIN diagnosis in the past three years were considered to be follow-up samples. Likewise, samples with ASCUS in the previous 15 months, with more severe cytological abnormalities or with a positive HPV test in the past 12 months were considered follow-up samples. Other study samples were considered primary samples. Reflecting routine cervical screening practice, primary samples included screening samples and a small proportion of samples taken by indication.

### Cytology

Cytology screening of LBC samples was performed routinely with a FocalPoint GS Imaging System (SlideWizard; BD, Burlington, NC, USA). Cytological outcomes were reported according to the Bethesda 2001 classification. They were classified as ASCUS, low-grade squamous intraepithelial lesions (LSIL), or high-grade squamous intraepithelial lesions or worse (≥HSIL) including atypical squamous cells – cannot exclude HSIL (ASC-H), atypical glandular cells (AGC), adenocarcinoma in situ (AIS) and carcinoma. Cytology was evaluated by cytoscreeners and pathologists without knowledge of the outcomes of HPV testing.

### Cobas HPV DNA Testing

For testing with cobas, 1 ml of the diluted material was aliquoted into a 13 ml round bottom test tube (Sarstedt, cat. no NC9018280). All tubes were labeled with the CPR number, barcode and the laboratory specimen identifier used for cytology testing. Subsequently, the tubes were stored at 2–8°C until testing according to the manufacturer’s protocol. No pre-treatment of SurePath samples was required. Extraction of DNA was undertaken on the cobas x 480 instrument [22]. Amplification and detection of high-risk HPV DNA were undertaken on the cobas z 480 analyzer. The real-time PCR platform amplified a sequence of 200 bp from the HPV L1 region using specific primers for the 14 targeted HPV genotypes, dNTP and the EagleZ05 DNA-Polymerase. Fluorescent TaqMan® probes were used for detection of the amplicons during PCR cycles. Amplification and detection of the 330 bp β-globin was used as an internal control of the testing processes. The end results were interpreted by the software as “negative”, “HPV16”, HPV18”, “other high-risk HPV”, or any combination of the latter three. Samples with invalid results were retested.

Additionally, we evaluated the intra-laboratory reproducibility of the cobas assay. For this study, we used samples tested as part of the pre-trial validation of the cobas equipment during which 10.8% of the cobas samples returned invalid. We selected 200 negative and 300 positive cobas samples from different batches, regardless of the women’s age, screening history or CT value. After the pre-trial validation, the company calibrated the instrumentation, resulting in a lower proportion of invalid samples (0.1%) for the duration of the trial.

### HC2 HPV DNA Testing

With HC2, the detection of 13 high-risk genotypes is done in a bulk fashion with a “Positive” or “Negative” sample result readout. The test has no internal control for sufficiency of test material. Samples were either pretreated manually with DNA denatured prior to testing according to the manufacturer’s protocol, or DNA was isolated and purified using the DSP AXpH DNA kit on the QIASymphony SP platform [23] (Qiagen, Hilden, Germany). Testing of these samples was performed on automated Rapid Capture® System (Qiagen, Hilden, Germany) using scripts depending on pretreatment. A minority of samples that were used for triage of women with ASCUS were denatured and tested manually as part of routine screening. Reading of results was measured using the DML 2000™ Instrument with the *digene* Hybrid Capture system version 2 software (DHCS v.2). Testing was done on cytology postquot material.

### Statistical Analysis

The outcomes of testing with cobas were reported hierarchically (HPV16>HPV18>other high-risk HPV>negative>inadequate). Positive cobas samples were defined in accordance with the manufacturer’s critical threshold (CT) values, being ≤40.5 for HPV16, ≤40.0 for HPV18, and ≤40.0 for other high-risk genotypes, and positive HC2 samples as those with a relative light unit per cut-off (rlu/co) value ≥1. Cytology was considered abnormal if ≥ASCUS was reported. Differences in the distributions of age, screening history, cytology, and HC2 outcomes between the included and excluded samples were tested with the χ^2^ test. The outcomes of the cobas testing were tabulated by the age of the women, screening history (primary vs. follow-up samples), cytology outcome, and the HC2 outcome. The trends in HPV positivity by age were tested with the Mantel-Haenszel χ^2^ test for trend, an 95% confidence intervals (CI) for relative risks were calculated by assuming that their logarithms were approximately normally distributed.

### Ethical Considerations

This study was designed as a quality development study, utilizing only residual material that would otherwise have been anonymized and discarded. According to Danish regulations of biomedical research, an ethical approval is not necessary for such studies, in accordance with "Guidelines about Notification etc. of a Biomedical Research Project to the Committee System on Biomedical Research Ethics, No 9154, 5 May 2011, section 2.5″.

## Results

Between the 5,072 samples available for testing with cobas, and the 1,186 samples that were excluded from the study, there were no significant differences in the distributions by age group (χ^2^ = 11.2, df = 6, P = 0.084), cytology outcome (χ^2^ = 3.58, df = 4, P = 0.466), or HC2 outcome (χ^2^ = 1.04, df = 1, P = 0.308). There was a small but significant difference between the included and the excluded samples in terms of the women’s screening history. In total, 87.0% of the included samples, and 89.9% of the excluded samples were primary samples (χ^2^ = 7.29, df = 1, P = 0.007).

The mean number of days between the arrival of the sample in the laboratory and storing was 2 (range: 1–5). The mean number of days between storing and testing was 16 (range: 1–62). The mean age of the women was 37.3 years (SD = 12.3, range: 16–89 years). In total, 162 (3.2%) samples were taken in women aged below the recommended start of screening (23 years), and 113 (2.2%) in women aged above the recommended ending of screening (65 years; Table 1). Reflecting a predominantly young female population in the catchment area of the laboratory, 3,063 samples (60.4%) were taken in women aged 23–39 years.

**Table 1:** Outcomes of the cobas assay for the 5,072 primary and follow-up samples included in the study by age, screening history, cytology, and Hybrid Capture 2 outcomes.

Total	Outcomes of HPV DNA testing with the cobas assay, N(%)						Positive outcomes on HPV DNA testing with the HC2 assay, N(%)^b^	Total
	HPV16^a^	HPV18^a^	Other 12 high-risk HPV genotypes^a^	Any 14 high-risk HPV genotype^a^	Negative	Inadequate		
**Total**	365 (7.2)	130 (2.6)	866 (17.1)	1,361 (26.8)	3,708 (73.1)	3 (0.1)	1,035 (20.4)	5,072 (100)
**Age (years)**								
16–22	30 (18.5)	10 (6.2)	62 (38.3)	102 (63.0)	60 (37.0)	0 (0)	92 (56.8)	162 (100)
23–29	201 (13.1)	57 (3.7)	405 (26.4)	663 (43.1)	874 (56.9)	0 (0)	507 (33.0)	1,537 (100)
30–39	87 (5.7)	41 (2.7)	244 (16.0)	372 (24.4)	1,154 (75.6)	0 (0)	266 (17.4)	1,526 (100)
40–49	30 (3.0)	17 (1.7)	100 (10.1)	147 (14.8)	846 (85.2)	0 (0)	112 (11.3)	993 (100)
50–59	7 (1.4)	2 (0.4)	30 (5.9)	39 (7.7)	466 (91.9)	2 (0.4)	37 (7.3)	507 (100)
60–65	7 (3.0)	3 (1.3)	14 (6.0)	24 (10.3)	210 (89.7)	0 (0)	14 (6.0)	234 (100)
>65	3 (2.7)	0 (0)	11 (9.7)	14 (12.4)	98 (86.7)	1 (0.9)	7 (6.2)	113 (100)
Average age (years)	30.4	31.2	31.7	31.3	39.5	59.7	31.0	37.3
**Screening history**								
Primary sample	287 (6.5)	106 (2.4)	688 (15.6)	1,081 (24.5)	3,331 (75.5)	1 (0.0)	822 (18.6)	4,413 (100)
Follow-up sample	78 (11.8)	24 (3.6)	178 (27.0)	280 (42.5)	377 (57.2)	2 (0.3)	213 (32.3)	659 (100)
**Cytology**								
Normal	273 (5.8)	102 (2.2)	697 (14.9)	1,072 (22.9)	3,599 (77.0)	2 (0.0)	728 (15.6)	4,673 (100)
ASCUS	19 (15.4)	9 (7.3)	50 (40.7)	78 (63.4)	45 (36.6)	0 (0)	79 (64.2)	123 (100)
LSIL	29 (20.3)	7 (4.9)	78 (54.5)	114 (79.7)	29 (20.3)	0 (0)	128 (89.5)	143 (100)
≥HSIL	44 (41.1)	12 (11.2)	39 (36.4)	95 (88.8)	12 (11.2)	0 (0)	97 (90.7)	107 (100)
≥ASCUS	92 (24.7)	28 (7.5)	167 (44.8)	287 (76.9)	86 (23.1)	0 (0)	304 (81.5)	373 (100)
Inadequate	0 (0)	0 (0)	2 (7.7)	2 (7.7)	23 (88.5)	1 (3.8)	3 (11.5)	26 (100)

Positive cobas outcomes were defined hierarchically, HPV16>HPV18>other 12 high-risk genotypes.

Abbreviations: ASCUS = atypical squamous cells of undetermined significance; HC2 = Hybrid Capture 2 assay; HPV = Human Papillomavirus; ≥HSIL: high-grade intraepithelial lesions or worse; LSIL = low-grade squamous intraepithelial lesions.

a Single and multiple infections combined.

b Eight samples were not tested with HC2.

### Prevalence of HPV Infection

Of the 5,072 samples, 22 (0.4%) had to be retested, with three (0.1%) remaining inadequate after retesting. Overall, 1,361 samples (26.8%) tested positive on cobas. Listing the HPV genotypes hierarchically based on oncogenicity for cervical cancer (HPV16>HVP18>12 other HR HPV genotypes>negative>inadequate), single or multiple infections with genotype 16 were detected in 365 (7.2%) samples; 130 (2.6%) samples showed single or multiple infections with HPV genotype 18 excluding any coinfections with HPV16; and the remaining 866 (17.1%) samples showed single or multiple infections with any of the remaining non-16/18 HPV genotypes targeted by the cobas assay.

For women targeted by the organized screening program (23–65 years), the overall proportion testing positive on the cobas assay decreased with age from 43.1% in women aged 23–29 years to 10.3% in women aged 60–65 years (Table 1; P for trend <0.0001). This trend was equally strong for HPV16, HPV18 and the bulk of other 12 high risk HPV genotypes (all P values for trend <0.0001). For comparison, the proportion of women aged 23–29 years testing positive on HC2 was 33.0%, whereas 17.4% tested positive at age 30–39 years, 11.3% at age 40–49 years, 7.3% at age 50–59 years, and 6.0% at age 60–65 years. The proportion of samples taken from women aged 16–22 years testing positive on the cobas assay was 63.0%. This could possibly be a consequence of presentation of women for a medical condition or of self-selection for screening as routine screening is not offered at this age. After age 60 years, the detection of HPV infections appeared to increase, however, the number of tested samples was small.

Only 373 (7.4%) samples were abnormal on cytology. Among women with normal cytology, 22.9% of the samples tested positive on cobas and 15.6% on HC2. Among women with ASCUS, these proportions were 63.4% for cobas and 64.2% for HC2; they were 79.7% and 89.5%, respectively, for LSIL; and 88.8% and 90.7%, respectively, for ≥HSIL.

**Figure 1:**
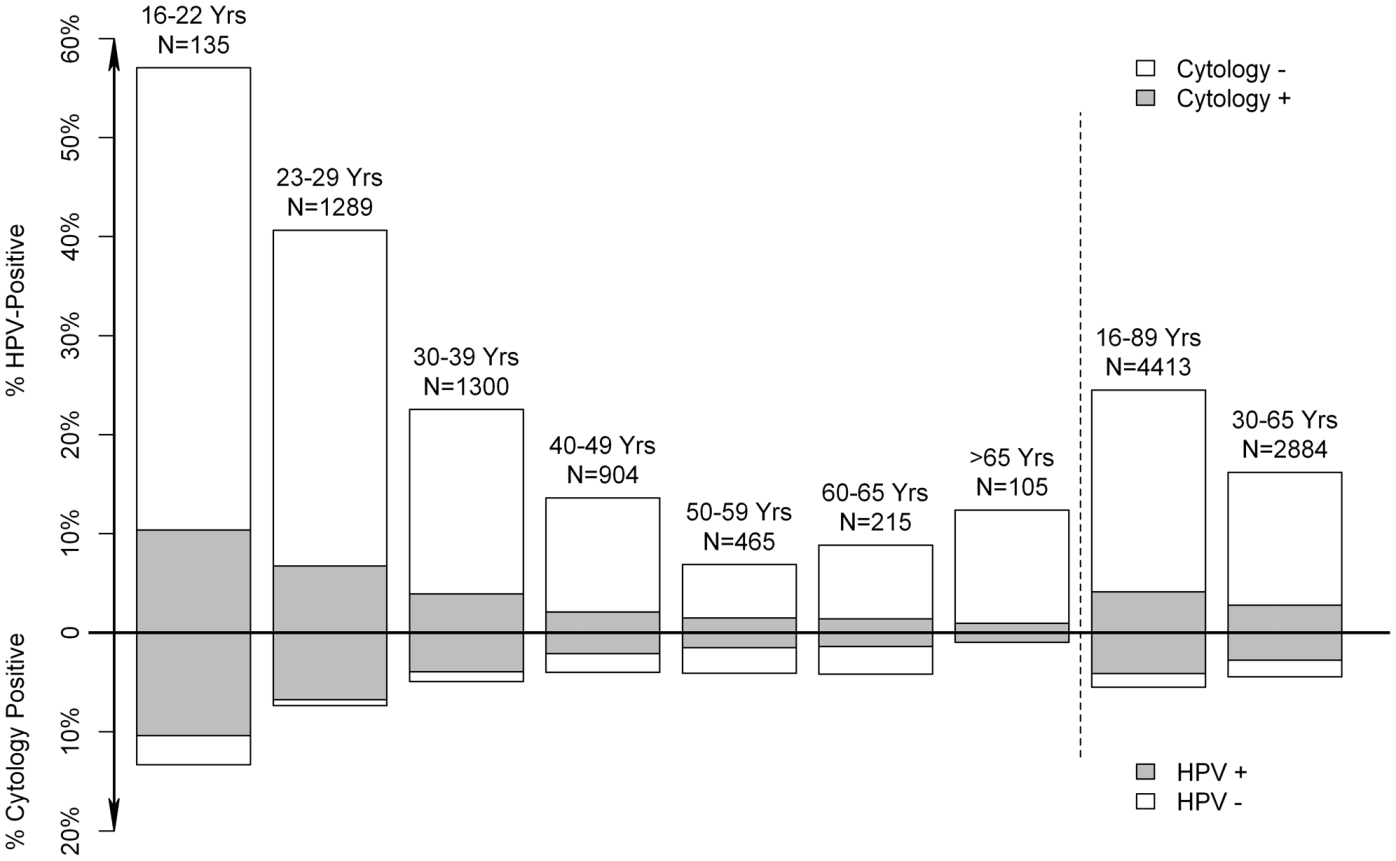
Primary cervical samples, by age and outcome of cytology and HPV testing with cobas.

These results were fairly similar for the subgroup of 4,413 primary tests (Table 2). In total, 1,081 (24.5%) of primary samples taken at any age would test positive if the cobas assay was used as the primary screening test (Figure 1). Now that cytology is used as a primary screening test, 242 (5.5%) samples tested positive. For all ages combined 182 (4.1%) of primary samples were positive on both the cobas assay and LBC. As the proportions of samples positive on the cobas assay varied considerably by age, so did the proportions of samples positive on cobas with normal cytology. Across all age groups, 898 of 4,413 (20.3%) samples were cobas HPV positive with normal cytology, but this proportion decreased from 33.9% in women aged 23–29 years to 18.6% at age 30–39 years (relative risk compared to 23–29 years: 0.55, 95% CI: 0.48–0.63), 11.5% at age 40–49 years (relative risk compared to 23–29 years: 0.34, 95% CI: 0.28–0.41), and 6.0% at age 50–65 years (relative risk compared to 23–29 years: 0.18, 95% CI: 0.13–0.24, P for trend <0.0001).

**Table 2:** The outcomes of the cobas assay for the 4,413 primary samples included in the study by age, screening history, cytology, and Hybrid Capture 2 outcomes.

Total	Outcomes of HPV DNA testing with the cobas assay, N(%)						Positive outcomes on HPV DNA testing with the HC2 assay, N(%)^b^	Total
	HPV16^a^	HPV18^a^	Other 12 high-risk HPV genotypes^a^	Any 14 high-risk HPV genotype^a^	Negative	Inadequate		
**Total**	287 (6.5)	106 (2.4)	688 (15.6)	1,081 (24.5)	3,331 (75.5)	1 (0)	822 (18.6)	4,413 (100)
**Age (years)**								
16–22	22 (16.3)	10 (7.4)	45 (33.3)	77 (57.0)	58 (43.0)	0 (0)	71 (52.6)	135 (100)
23–29	157 (12.2)	46 (3.6)	321 (24.9)	524 (40.7)	765 (59.3)	0 (0)	408 (31.7)	1,289 (100)
30–39	65 (5.0)	32 (2.5)	196 (15.1)	293 (22.5)	1,007 (77.5)	0 (0)	205 (15.8)	1,300 (100)
40–49	28 (3.1)	14 (1.5)	81 (9.0)	123 (13.6)	781 (86.4)	0 (0)	94 (10.4)	904 (100)
50–59	6 (1.3)	2 (0.4)	24 (5.2)	32 (6.9)	432 (92.9)	1 (0.2)	30 (6.5)	465 (100)
60–65	6 (2.8)	2 (0.9)	11 (5.1)	19 (8.8)	196 (91.2)	0 (0)	8 (3.7)	215 (100)
>65	3 (2.9)	0 (0)	10 (9.5)	13 (12.4)	92 (87.6)	0 (0)	6 (5.7)	105 (100)
Average age (years)	30.8	31.0	31.9	31.5	39.8	54.0	31.0	37.8
**Cytology**								
Normal	234 (5.6)	90 (2.2)	574 (13.8)	898 (21.6)	3,251 (78.3)	1 (0)	625 (15.1)	4,150 (100)
ASCUS	13 (15.3)	6 (7.1)	35 (41.2)	54 (63.5)	31 (36.5)	0 (0)	54 (63.5)	85 (100)
LSIL	14 (16.1)	5 (5.7)	48 (55.2)	67 (77.0)	20 (23.0)	0 (0)	79 (90.8)	87 (100)
≥HSIL	26 (37.1)	5 (7.1)	30 (42.9)	61 (87.1)	9 (12.9)	0 (0)	62 (88.6)	70 (100)
≥ASCUS	53 (21.9)	16 (6.6)	113 (46.7)	182 (75.2)	60 (24.8)	0 (0)	195 (80.6)	242 (100)
Inadequate	0 (0)	0 (0)	1 (4.8)	1 (4.8)	20 (95.2)	0 (0)	2 (9.5%)	21 (100)

Positive cobas outcomes were defined hierarchically, HPV16>HPV18>other 12 high-risk genotypes.

Abbreviations: ASCUS = atypical squamous cells of undetermined significance; HC2 = Hybrid Capture 2 assay; HPV = Human Papillomavirus; ≥HSIL: high-grade intraepithelial lesions or worse; LSIL = low-grade squamous intraepithelial lesions.

a Single and multiple infections combined.

b Six samples were not tested with HC2.

### Intra-laboratory Reproducibility of the Cobas Assay

Among the 300 samples that tested positive on the initial cobas run, 272 (90.7%) tested positive also in the second run. Among the 28 (9.3%) samples that changed from positive to negative, the average CT value was 38.0 (range: 16.3–40.1) in the first run, and 19 were uniquely positive for “other high-risk genotypes”. Among the 200 samples that tested negative on the initial cobas run, 196 (98.0%) tested negative in the second run, whereas 4 (2.0%) changed the outcome from negative to positive. The average CT value of the latter samples was 39.3 (range: 38.8–40.0) in the second run, suggesting weakly positive HPV samples.

## Discussion

### Main Findings

In Copenhagen, where the background risk of cervical cancer has been high, 27% of women tested positive for the bulk of 14 high-risk HPV genotypes included in the cobas assay. Even in samples without recent cervical abnormalities, 24% tested positive. As expected, this was considerably higher than the 7% and 5% of women, respectively, with abnormal cytology. The proportions of women testing positive on cobas decreased from 43% (41%) in women aged 23–29 years to 10% (9%) in women aged 60–65 years. About two-thirds of the positive cobas tests were due to genotypes other than HPV16 and HPV 18. This is not unusual, as infections with these HPV genotypes are frequent in women with normal cytology or with low-grade CIN [24], [25], though they only cause about one-third of cervical cancers [9].

In line with the higher prevalence of HPV genotypes 16 and 18 in high-grade lesions, 52% of cytological ≥HSIL in our study showed an infection with these two genotypes. However, we found that 11% of samples with ≥HSIL on cytology (0.2% of all samples) tested negative on the cobas assay. In the Danish screening program, women with ≥HSIL are currently referred for colposcopy without HPV DNA triage. We will monitor them through linkage with the Pathology Data Bank to determine whether HPV triage could be used to reduce false-positive referrals for colposcopy.

More surprising was the fact that the proportion of positive samples was 27% for cobas versus 20% for HC2. Some of this difference may be due to the differences in the designs of the assays. While the cobas was designed to detect 14 HPV types, HC2 was designed to detect 13 HPV types. Furthermore, cobas is a real-time PCR-based L1 DNA assay, whereas HC2 is an RNA to DNA hybridization assay.

Although using samples from the pre-trial validation of the cobas instrumentation, the intra-laboratory reproducibility of negative cobas results was high. The 2.0% of the initially negative samples that tested positive in the second run showed very high average CT values. This is reassuring given the on-going international discussions on extending the screening interval. However, the 90.7% reproducibility of the initially positive cobas results suggests that the calibration of the probe for “other high-risk genotypes” is not highly robust. In a Dutch validation study using Universal Collection Medium, the intra-laboratory positive reproducibility of the cobas assay was 97.3% [26], suggesting that the choice of the SurePath media could in part explain the low positive reproducibility of the cobas assay in our study.

### Strengths and Weaknesses

Our study was population-based and used consecutive samples from a large public laboratory. Nineteen percent of the selected samples had to be discarded because only small amounts of residual material were available for the study. There was no significant difference between the included and the excluded samples in terms of the age of the women, cytology interpretation, and the HC2 outcome. All samples were tested in one laboratory by the same staff that is involved in routine screening. The proportion of women aged 25–64 years without recent abnormalities who tested positive on the HC2 assay, 16%, was similar to that observed in an earlier study covering the same catchment area, approximately 17% [27]. The 5,072 tested samples can therefore be considered as highly representative for the study population.

The cobas assay, furthermore, functioned well on the diluted SurePath media, as only 3 (0.1%) out of 5,072 samples had an inadequate outcome. Our study is the first to report the cobas outcomes based on SurePath samples. SurePath has an estimated market share in England of about 55%, 70% in Denmark (Ole Jakobsen, Axlab, personal communication, 2012), and about 30% in the USA [28]. Previous studies of the cobas assay used samples stored in PreservCyt [11], [14]–[17], [29]–[32], specimen transport medium [18], or universal collection medium [26]. SurePath contains 0.1% formaldehyde as a fixative which may eliminate enzymatic activity, so the outcomes of HPV testing may vary by transport media.

Following the standard recommendations within the Danish cervical screening program, women in our study population with abnormal cytology were referred for colposcopy or for repeated testing. As a follow-up to the current study, we will additionally invite women with positive HPV tests and normal cytology for repeated testing in 12 months. The standard measures of sensitivity and specificity of the screening tests will be reported once the histological follow-up of the study population has been completed.

### Comparison with Previous Studies

The cobas assay was FDA-approved based on the ATHENA study which included women presenting for routine screening [11]. The proportion of women aged 25–59 years with abnormal primary cytology was slightly higher in ATHENA, 6.5%, than in our study, 5.0%, standardized to match the age distribution in the ATHENA study. This difference derived from women aged below 50 years, whereas no difference was found for women aged 50–59 years. The higher cytology abnormality proportion in the USA compared to Denmark could be explained by differences in the background risk of cervical cancer, and in the interpretation of cytology, especially in pre-menopausal women. On the other hand, the age-standardized proportion of women with positive cobas samples was lower in ATHENA than in our study, 10.8% vs. 19.4%, respectively, again as a result of differences among women below age 50 years. The higher HPV-positivity proportion in our study could reflect technical differences deriving from comparison of PreservCyt with SurePath samples, or it could reflect a higher prevalence of HPV infection. Concerning technical differences, one would then expect equal differences across age-groups, but this was not the case. Therefore, the difference is most likely a consequence of a higher prevalence of HPV infections in the Danish compared to the USA population. Consequently, for women aged 25–59 years the gap between the proportion of positive cobas samples and the proportion of abnormal cytology was more than twice as large in our study as in ATHENA (3.9 vs. 1.7, respectively). This stresses the need for local trial data before decisions on future screening tests are taken on regional or national level.

Several studies evaluated the use of the cobas assay among women referred for colposcopy because of abnormal cytology. The Predictors 2 study compared the cobas assay to three other HPV DNA assays (HC2, Abbott RealTi*m*e, and BD HPV test), two HPV mRNA assays (Gen-Probe APTIMA, Norchip Proofer), and one immunocytochemical assay (MTM CINtec p^16INK4a^) in 1,099 women [14]. Cobas was among the most sensitive tests for ≥CIN3, however not among the most specific for ≥CIN2. In the “Early Evaluator Program” study undertaken on a convenience sample of 1,360 women from Spain, France and Italy primarily presenting for follow-up of an earlier abnormality, 87% of samples were concordant on cobas and HC2. However, among the discordant samples those negative on cobas were more likely to show infections with low-risk HPV genotypes, whereas among those positive on cobas a larger proportion showed infections with high-risk HPV genotypes [32]. This distinction was corroborated in a study of 1,852 women from the USA retested one year after positive HC2 results [18], but not in 472 women presenting for routine LBC in Canada [29].

### Implications for Screening

Because of the high proportions of young women testing positive, HPV DNA testing has been proposed from age 30 or 35 years onwards. In our study, primary samples (including a small proportion of samples taken by indication, reflecting the routine running of the Danish screening program) of 16.2% of women aged 30–65 years tested positive on cobas, and 2.8% of women had both a positive cobas test and abnormal cytology (Figure 1). In contrast, 4.4% of women at this age had abnormal primary cytology. Based on historical data from the laboratory, 1.3% of screened women had abnormal cytology and ≥CIN3 detected in the follow-up. Assuming that HPV DNA testing is 32% more sensitive for ≥CIN3 than cytology [1], about 1.7% of women would be expected to have a positive cobas test and a ≥CIN3 lesion. Thus, with primary HPV DNA testing with the cobas assay, about 14.5% (16.2% positive on cobas −1.7% as the expected ≥CIN3 detection rate) of screened women would have a ≥CIN3 false-positive test, whereas with cytology-based screening this is now about 3.1% ( = 4.4% abnormal cytology −1.3% as the expected ≥CIN3 detection rate on cytology).

Much work has been done to define optimal criteria for referral of HPV-positive women for colposcopy [10], [33]. In our study, 2.8% of women aged 30–65 years could be immediately referred for colposcopy if cytology ≥ASCUS was set as the threshold for HPV-positive samples. This means that the remaining 13.4% ( = 16.2% positive on cobas −2.8% positive on cobas and cytology) of women would be followed-up initially with repeated testing. However, this proportion might vary across settings. In the ATHENA trial, it was 6.7% for women above age 30 years [13].

Reducing the extent of repeated testing is a priority issue for introducing HPV DNA-based primary screening. Countries with a high prevalence of HPV infection such as Denmark should therefore proceed to HPV-based primary cervical screening with caution because the extent of follow-up testing and diagnostics could be enormous whether or not triage is used to refer women for colposcopy.

### Conclusions

Our study showed a 27% high-risk HPV positivity proportion when unselected women from Copenhagen were tested with the cobas assay, and 20% when tested with HC2. These positivity rates were far above the 7% abnormality rate found in routine cytology screening. Even in women without recent abnormalities aged ≥30 years these percentages were 16% and 4%, respectively.
